# Estimation of Genetic Parameters for Semen Quality Traits in Large White and Landrace Boars

**DOI:** 10.3390/ani16213425

**Published:** 2026-10-29

**Authors:** Xiwei Cui, Yanyan Li, Yuanyuan Guo, Xiaotian Qiu, Chousheng Liu, Shengyue Gao, Kexin Sun, Peng Gai, Xuankai Huang, Peng Zuo, Zhipeng Wang

**Affiliations:** 1 College of Animal Science and Technology, Northeast Agricultural University, Harbin 150000, China; 19803350379@163.com (X.C.);; 2 College of Information Engineering, East University of Heilongjiang, Harbin 150066, China; 3 National Animal Husbandry Service, Beijing 100125, China; 4 Branch of Animal Husbandry and Veterinary, Heilongjiang Academy of Agricultural Sciences, Qiqihar 161005, China; 5 Heilongjiang DBN Food Science and Technology Group Co., Ltd., Harbin 150000, China; 6 College of Smart Science and Engineering, Northeast Agricultural University, Harbin 150000, China

**Keywords:** boar semen trait, repeatability model, random regression model, genetic parameters

## Abstract

Artificial insemination is used widely in the swine industry, resulting in increasing focus on the traits of boar semen and methods for their genetic improvement. This study analyzed 21,160 phenotypic records of fresh semen derived from 492 Large White boars and 231 Landrace boars. The semen traits included the semen volume (VOL), sperm concentration (CON), sperm motility (MOT), and abnormal sperm rate (ABN). Single-trait and multi-trait repeatability models were used to assess the overall genetic parameters of semen traits, while random regression models were applied to examine trends in heritability changes over the collection period. In addition, multi-trait repeatability models were also used to estimate genetic and phenotypic correlations among the traits. The single-trait repeatability model indicated heritability estimates of VOL, MOT, CON, and ABN of 0.23, 0.09, 0.22, and 0.07, respectively, for Large White boars, with corresponding values for Landrace boars of 0.42, 0.06, 0.16, and 0.17. The respective values calculated by the multi-trait repeatability model were 0.23, 0.08, 0.22 and 0.09 for Large White boars and 0.43, 0.06, 0.20 and 0.17 for Landrace boars. Sperm motility, sperm concentration, and abnormal sperm rate showed low-to-moderate heritability. Semen volume showed moderate heritability in Large White boars and high heritability in Landrace boars. The random regression results indicated heritability estimates for VOL, MOT, CON, and ABN of 0.24–0.53, 0.05–0.08, 0.13–0.23, and 0.03–0.31, respectively, for Large White boars and 0.08–0.31, 0.02–0.15, 0.26–0.53, and 0.12–0.17 for Landrace boars. Significant negative genetic correlations were found between VOL and CON in both breeds (−0.54 for Large White and −0.73 for Landrace), and between VOL and MOT in Landrace. These findings will facilitate more precise selection to improve boar semen quality and reproductive efficiency in the swine industry.

## Simple Summary

In this study, we estimated the genetic parameters for semen volume, sperm concentration, sperm motility, and abnormal sperm rate in Large White and Landrace boars. The results showed that sperm motility had low heritability, sperm concentration and abnormal sperm rate showed moderate heritability, and semen volume displayed moderate to high heritability. Importantly, we found age-dependent trends of the two breeds. We also identified genetic correlations among traits, for example, a negative correlation between semen volume and sperm concentration, and a positive correlation between sperm motility and sperm concentration. These findings provide valuable guidance for pig breeders to design more precise selection and help to improve semen quality and reproductive efficiency in the swine industry.

## 1. Introduction

The genetic makeup has a crucial impact on production performance in the pig industry [1,2,3]. The selection of breeding boars is based on growth [4,5], carcass [6,7], and reproductive traits [8,9]. Semen quality is a critical determinant of boar reproductive performance, encompassing semen volume, sperm motility, sperm concentration, and abnormal sperm rate. Semen volume and sperm concentration reflect the ejaculatory capacity of the boar’s testes, while sperm motility and the abnormal sperm rate influence overall semen quality, fertilization capacity, and sow conception rates [10]. Reduced semen quality can also significantly shorten boar lifespans [11]. Therefore, estimation of the genetic parameters associated with boar semen is important for both scientific selection and ensuring reproductive efficiency in boars. Although several studies have estimated the genetic parameters associated with boar semen traits [12,13], there are marked discrepancies among different investigations. Most studies [14,15,16,17] have reported that semen volume and sperm concentration show moderate heritability. Martin et al. [18] obtained heritability estimates of 0.32 and 0.35 for semen volume and sperm concentration, respectively, both of which fall into the high heritability category. Most studies [13,15,19,20] have reported that sperm motility and the abnormal sperm rate represent traits with low-to-moderate heritability. However, Zhang et al. [21] found that sperm motility was highly heritable, while both Zhang et al. and Wolf et al. [16] observed that abnormal sperm rates were also highly heritable. In terms of genetic correlations, studies by both Martin et al. [18] and Wolf et al. [14] demonstrated a positive association between semen volume and sperm motility, although Li et al. [22] observed that the correlation varied in terms of pig breed, being positive in Landrace and Yorkshire pigs and negative in Duroc pigs. There is a clear need for systematic research on the estimation of genetic parameters for boar semen traits across different populations and various environmental conditions.

Semen traits in boars represent longitudinal observational data, as the traits change dynamically with the boar’s age [23], and their physiological basis is related to testicular development and hormonal regulation. Boars reach sexual maturity at approximately 9–12 months of age, during which GnRH, FSH, and LH secretion increases, testosterone levels rise, and semen quality improves rapidly. This is followed by a plateau phase, and after 24 months of age, boars enter the aging stage, characterized by testicular degeneration, accumulated oxidative stress, and decreased testosterone, leading to a decline in semen quality [23,24]. These dynamic changes make it difficult for animal models to fully capture the trend of trait variation over time and differences in measurement conditions across time points. To address the issue of assessing heritability that changes over time, Schaeffer proposed the day-of-measurement model in the early 1990s [25]. This model provides a more accurate estimation of genetic parameters of traits as it considers the temporal dimension. While this model has been widely applied to examinations of lactation traits in dairy cows [26] and growth traits in pigs [27,28], investigation of porcine semen traits remains relatively limited.

In this study, both single-trait and multi-trait repeatability models were used to estimate the genetic parameters of semen traits in Large White and Landrace boars. Random regression models were used to assess trends of heritability changes over age at semen collection (days). The findings of this study provide a theoretical basis for improving semen quality traits in both Large White and Landrace pig populations.

## 2. Materials and Methods

### 2.1. Study Population

The data for this study were obtained from the breeding farm located in Heilongjiang Province, China. This dataset includes 13,413 fresh semen quality feature records from 492 Large White pigs and 7747 fresh semen quality feature records from 231 Landrace pigs. All boars were born on a single Great Grand Parent farm. They were transferred to 4 AI stations after genetic evaluation. Semen quality trait data was collected from 4 AI stations. Large White boars were born between 2016 and 2022, and their semen collection records spanned from 2017 to 2024. Landrace boars were born between 2016 and 2022, with semen collection records from 2016 to 2024. The semen collection frequency was approximately once per week, with collections performed year-round, covering all four seasons, and the boars were group-housed. The pedigree was traced back three generations and included the phenotyped boars and their ancestors, but not their descendants. For Large White, 492 phenotyped boars were included, and the pedigree comprised 3638 animals, with birth years ranging from 2007 to 2022; the phenotyped boars were born between 2016 and 2022. For Landrace, 231 phenotyped boars were included, and the pedigree comprised 2577 animals, with birth years ranging from 2010 to 2022; the phenotyped boars were born between 2015 and 2022.

### 2.2. Data Collection and Quality Control

Semen was collected in pneumatic tubes and was transported to the laboratory, where laboratory personnel used the Computer-Aided Sperm Analysis (CASA) system to analyze sperm quality and were responsible for compiling and archiving the test results. The analyzed traits included semen volume (VOL), sperm motility (MOT), sperm concentration (CON), and abnormal sperm rate (ABN). MOT refers to total sperm motility (i.e., the percentage of motile spermatozoa, including both progressive and non-progressive motile sperm). ABN refers to the percentage of spermatozoa with morphological abnormalities, including abnormalities of the head, midpiece, and tail. Semen volume was determined by weighing on an electronic balance, while MOT, CON, and ABN were assessed using the Magapor Gesipor 3.0 CASA system.

The criteria for data inclusion were as follows: (1) boars aged between 6 and 29 months; (2) records with VOL values between 30 and 600 mL; (3) records with MOT values between 10.00 and 99.00%; (4) records with CON values between 0.01 × 10^8^ and 10 × 10^8^/mL; (5) records with ABN values of 0–90.00%. The exclusion criteria were (6) samples from boars with ≤3 semen records and (7) records where the interval between consecutive collections was <2 or >60 days. Boars of the same breed were grouped according to the month of semen collection (i.e., age in months). Semen records from the same month were assigned to the same group, and any value outside three standard deviations from the group mean was excluded.

Since the abnormal sperm rate (ABN) did not follow a normal distribution, it was normalized using the Box–Cox transformation. Because ABN contained zero values (minimum = 0), a constant c = 1 × 10^−6^ was added to all ABN records before transformation, so that ABN = 0 became 1 × 10^−6^. The optimal Box–Cox parameters estimated by maximum likelihood were λ = 0.305 for Large White and λ = 0.188 for Landrace. The transformed ABN data approximately followed a normal distribution. Therefore, ABN was analyzed using the transformed data in subsequent analyses, and all reported heritabilities and genetic correlations involving ABN are expressed on the transformed scale.

### 2.3. Statistical Analysis

#### 2.3.1. Model for Estimating Variance Components

The following models were used to estimate the genetic parameters for the four semen traits:

(1) Single-trait repeatability model, as represented by the formula:

y = Xb+ Za + Wpe + e

where y is a vector of phenotypic records for each semen quality trait; b represents a vector of fixed effects, including the herd-year-season effect (4 AI stations, 2016–2024 year, and March to May is spring, June to August is summer, September to November is autumn, and December, January, and February is winter), age at semen collection (in months, treated as a continuous covariate), and the collection interval (in days, treated as a continuous linear covariate); a is a vector representing the additive of individual genetic effects, with a ~ N (0, Aσ_a_^2^), where σ_a_^2^ is the additive genetic variance and A is the pedigree-based relationship matrix; pe is a vector associated with random permanent environmental effects, with pe ~ N (0, Iσ_pe_^2^), where σ_pe_^2^ is the permanent environmental variance; e is a vector of residual effects, with e ~ N (0, Iσ_e_^2^), where I is the identity matrix and σ_e_^2^ is the residual variance; and X, Z, and W are incidence matrices corresponding to b, a, and pe, respectively.

(2) Multi-trait repeatability model, as represented by the formulae:

y1=X1b+Z1a+W1pe +e1 y2=X2b+Z2a+W2pe +e2 y3=X3b+Z3a+W3pe +e3 y4=X4b+Z4a+W4pe +e4

where y_1_, y_2_, y_3_, and y_4_ represent the vector of observed values for individual VOL, CON, MOT, and ABN values, respectively. The multi-trait repeatability model enables simultaneous analysis of the four semen traits to obtain genetic and phenotypic correlations between the different traits. The remaining letters are as described above for the single-trait repeatability model.

(3) Random regression model, as represented by the following equation:

$$y_{\mathrm{ijt}}=s_{i}+g_{j}+\sum_{l=0}^{\mathrm{nr}}\varphi_{n}\left(w_{t}\right)a_{k}+\sum_{l=0}^{\mathrm{nr}}\varphi_{n}\left(w_{t}\right)Pe_{k}+e_{\mathrm{ijt}}$$

where *y_ijt_* is the observation for the kth individual at time t; s_i_ is the fixed effect, including the herd-year-season effect; *g_j_* is a continuous linear covariate, representing only the interval between semen collection; age at semen collection was modeled using Legendre polynomials only in the random regression part of the RRM for the additive genetic and permanent environmental effects; *a_k_* and *Pe_k_* are random regression coefficients for the additive genetic and permanent environmental effects, respectively; the terms φ_n_(w_t_) correspond to Legendre polynomials evaluated at standardized time w_t_ (−1 ≤ w_t_ ≤ 1), and the residual is given by *e_ijt_*.

In the random regression model, age at semen collection was defined as the age of the boar at semen collection in days. To satisfy the definition domain of Legendre polynomials, age was linearly rescaled to the interval [−1, 1] using min–max normalization:

$$t_{\mathrm{norm}}=-1+2\times \frac{\mathrm{age}-{\mathrm{age}}_{\min}}{{\mathrm{age}}_{\max}-{\mathrm{age}}_{\min}}$$

where *age_min_* is the minimum sperm collection age, *age_max_* is the maximum sperm collection age. For Large White boars, *age_min_* = 183 and *age_max_* = 870 days; for Landrace boars, *age_min_*= 185 and *age_max_* = 870 days.

Residual variance was assumed to be homogeneous across all age groups, and a uniform residual variance was estimated for data over the whole age range of semen collection. This assumption was adopted because the number of records varies among different age groups and there are limited records at both ends of the age range. Heterogeneous residual variance structures require additional parameters, which may lead to convergence issues and overfitting [29,30]. For the covariance structure of random regression coefficients, unstructured covariance matrices were adopted for both additive genetic and permanent environmental effects. Specifically, all variances and covariances among regression coefficients of different orders (i.e., lg0, lg1, lg2, …) were freely estimated without artificial constraints. This represents the standard and most-flexible modeling strategy for random-regression analyses based on Legendre polynomials in animal genetic evaluation.

#### 2.3.2. Estimation of Genetic Parameters

DMU software (v6-R5.2-EM64T) [31,32] was employed, and the average information (AI) method and the expectation-maximization (EM) algorithm were applied to estimate the variance components for the various random effects in the model. The convergence criteria of the models were set to 1 × 10^−7^.

The formulae for calculating heritability and repeatability using the repeatability models were as follows:

$$h^{2}=\frac{\sigma_{a}^{2}}{\sigma_{a}^{2}+\sigma_{\mathrm{pe}}^{2}+\sigma_{e}^{2}},\;r_{e}=\frac{\sigma_{a}^{2}+\sigma_{\mathrm{pe}}^{2}}{\sigma_{a}^{2}+\sigma_{\mathrm{pe}}^{2}+\sigma_{e}^{2}}$$

where h^2^ represents heritability; σ_a_^2^ represents additive genetic variance; σ_pe_^2^ is the variance of the permanent random environmental effect; σ_e_^2^ represents the variance of the random residuals, and re is the repeatability.

The formula for calculating heritability at time t using the random regression model was as follows:

$${h_{t}}^{2}=\frac{\sigma_{a}^{2}\left(t\right)}{\sigma_{a}^{2}\left(t\right)+\sigma_{\mathrm{pe}}^{2}\left(t\right)+\sigma_{e}^{2}\left(t\right)}$$

where h_t_^2^ is the heritability at a specific time point; σ_a_^2^(t) represents additive genetic variance; σ_pe_^2^(t) is the variance of the permanent random environmental effect; and σ_e_^2^(t) represents the variance of the random residuals, with t indicating a specific time point.

The genetic correlation calculation formula:

$$r_{A}=\frac{\mathrm{cov}(a_{1},a_{2})}{\sigma_{a1}\sigma_{a2}}$$

where r_A_ represents the genetic correlation between trait 1 and trait 2, *cov*(*a*_1_, *a*_2_) denotes the genetic covariance between the two traits, and *σ_a_*_1_ and *σ_a_*_2_ stand for the genetic standard deviations of trait 1 and trait 2, respectively.

The phenotypic correlation calculation formula:

$$r_{P}=\frac{\mathrm{cov}(p_{1},p_{2})}{\sigma_{p1}\sigma_{p2}}$$

where r_P_ represents the phenotypic correlation between trait 1 and trait 2, *p*_1_ and *p*_2_ represent the phenotypic variations of trait 1 and trait 2, respectively; *cov*(*p*_1_, *p*_2_) represents the phenotypic covariance between the two traits, i.e., *cov*(*p*_1_, *p*_2_) = *cov*(*a*_1_, *a*_2_) + *cov*(*e*_1_, *e*_2_), *cov*(*e*_1_, *e*_2_) represents the residual covariance between two traits; σ_p1_ and σ_p2_ represent the phenotypic standard deviations of trait 1 and trait 2, respectively.

### 2.4. Model Comparison

Model selection for the random effects was based on the Akaike information criterion (AIC) [33]. This can be defined as follows:

AIC = −2log(L) + 2p

Similarly, the other most popular information criterion used was the Bayesian information criterion (BIC) [34], which puts a more stringent penalty on extra parameters and accounts for model uncertainty. The BIC is defined as follows:

BIC = −2log(L) + p × log(n)

where L represents the maximum likelihood function of the model; p is the total number of independent parameters in the model; and n is equal to the total number of records used in the analysis.

In addition to AIC and BIC, we also evaluate the stability of parameter estimates for all convergent stochastic regression models by calculating the ratio of the standard error of each variance and covariance component to the estimated absolute value (SE/| estimate|). If the SE/|Estimate| ratio of a parameter exceeds 1.0, it is considered unreliable. Among the remaining models, select the simplest model with acceptable AIC and BIC values and all SE/|Estimate| ratios below 1.0 as the final model for each trait.

## 3. Results

### 3.1. Descriptive Statistical Analysis of Phenotypic Data

After quality control and application of the inclusion and exclusion criteria, 1483 semen phenotype records of Large White boars were excluded, accounting for 9.96% of the original data, resulting in 13,413 semen phenotype records from 492 Large White boars. Similarly, for Landrace boars, 1436 semen phenotype records were excluded, accounting for 15.64% of the original 9183 records, and finally 7747 semen phenotype records of 231 Landrace boars were retained.

The descriptive statistics for the semen traits of Large White and Landrace boars are shown in Table 1. The mean VOL values for Large White and Landrace boars were 211.10 mL and 221.21 mL, respectively, while the mean CON values were 3.43 × 10^8^/mL and 3.57 × 10^8^/mL.

### 3.2. Estimation of the Heritability and Repeatability of Semen Quality Traits

#### 3.2.1. Single-Trait Repeatability Model

Table 2 shows the variance components and heritability of semen traits in Landrace and Large White boars, as determined using the single-trait repeatability model. The heritability values for VOL, MOT, CON, and ABN in Large White boars were 0.23 ± 0.05, 0.09 ± 0.03, 0.22 ± 0.05, and 0.07 ± 0.04, respectively. The estimated heritability of VOL in Landrace boars was 0.42 ± 0.10, which was higher than that in Large White boars. The heritability estimates of other semen traits were comparable between the two breeds, with MOT, CON, and ABN values of 0.06 ± 0.03, 0.16 ± 0.07, and 0.18 ± 0.07 in Landrace boars, respectively.

#### 3.2.2. Multi-Trait Repeatability Model

The variance components and heritability estimates of semen traits in Large White and Landrace boars determined using the multi-trait repeatability model are presented in Table 3. For Large White boars, the heritability estimates for VOL, MOT, CON, and ABN were 0.23 ± 0.05, 0.08 ± 0.03, 0.22 ± 0.05, and 0.09 ± 0.04, respectively; for Landrace boars, the corresponding estimates were 0.43 ± 0.10, 0.06 ± 0.03, 0.20 ± 0.07, and 0.17 ± 0.07.

#### 3.2.3. Random Regression Models

##### Selection of the Order of the Random Regression Model

In this study, Legendre polynomials of orders 0 to 3 were nested into the additive genetic effects and permanent environmental effects. Each trait was assessed using 16 different order combinations. The number of parameters for all models ranges from 5 to 21. In the converged models, different combinations were evaluated using the AIC and BIC criteria as well as the ratio of standard error to the absolute estimate (SE/|estimate|) to determine the optimal random regression models for different semen traits in the two breeds. The results are shown in Table 4. For Large White boars, the optimal additive genetic orders were 1, 0, 0, and 1 for VOL, MOT, CON, and ABN, respectively, while the corresponding permanent environmental orders were 2, 1, 1, and 3. For Landrace boars, the optimal additive genetic orders were 0, 1, 1, and 0 for VOL, MOT, CON, and ABN, respectively, and the corresponding permanent environmental orders were 1, 2, 0, and 1.

##### Heritability Estimation in Random Regression Models

**Figure 1:**
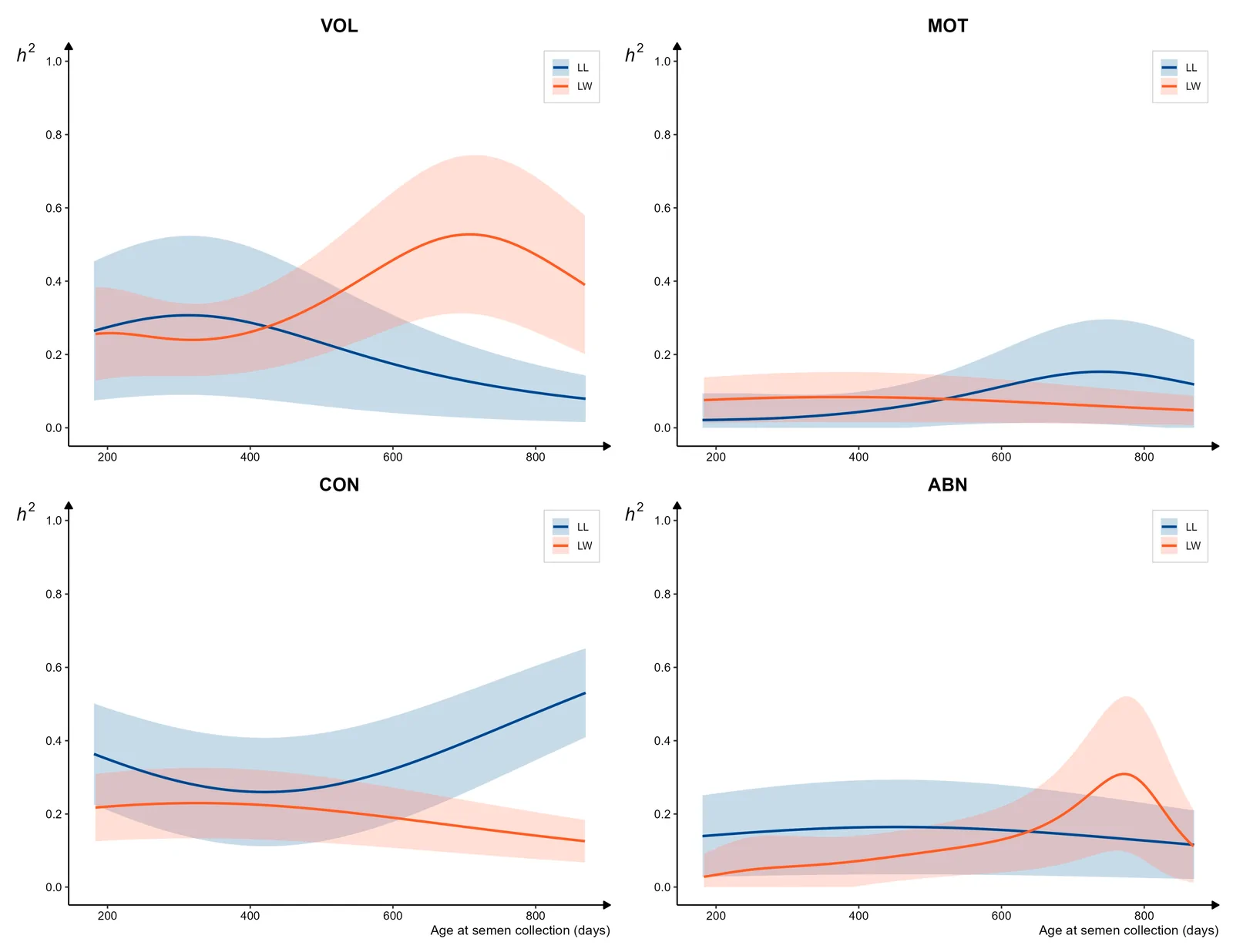
Heritability curves calculated using the random regression model. Shaded areas represent 95% confidence intervals.

The heritability ranges and corresponding standard errors for the optimal model combination of semen traits in Landrace and Large White boars are detailed in Table 5. For Large White boars, the heritability ranges of VOL, MOT, CON, and ABN were 0.24–0.53, 0.05–0.08, 0.13–0.23, and 0.03–0.31, respectively, while the corresponding heritability ranges for the four semen traits in Landrace boars were 0.08–0.31, 0.02–0.15, 0.26–0.53, and 0.12–0.17. The shadow in Figure 1 represents the 95% confidence interval.

The trends of change in heritability over time for the two breeds are shown in Figure 1. In Large White boars, VOL heritability increased rapidly in early life, reached a relatively high level around 600 days of age, then declined and rose again at older ages. MOT remained generally low with limited variation and declined at later ages. CON gradually increased in early and middle stages but decreased at the oldest ages. ABN was generally low, relatively stable early, temporarily increased around 900 days of age, and then dropped rapidly. Overall, some traits (e.g., VOL and CON) showed increasing heritability before adulthood but declined at older ages.

In Landrace boars, the trends differed. VOL slightly decreased early, then continuously increased and stabilized in adulthood. CON increased steadily with age and reached a higher level in adulthood. MOT decreased early, remained low, then gradually increased with age, reaching a higher level at older ages. ABN generally increased with age, was higher in adulthood, and then slightly declined. Overall, most semen traits in Landrace boars showed an increasing trend in heritability with age, particularly for CON and MOT, whereas VOL and ABN tended to stabilize or slightly decline at older ages.

### 3.3. Estimated Genetic Correlations for Semen Quality Traits

The multi-trait repeatability model was used to estimate the genetic and phenotypic correlations among semen traits in Large White and Landrace boars. Table 6 presents the results for Large White boars, showing negative genetic correlations between VOL and CON (−0.54 ± 0.13), VOL and MOT (−0.03 ± 0.23), and MOT and ABN (−0.16 ± 0.31), a positive genetic correlation between MOT and CON (0.34 ± 0.19), and positive genetic correlations for all remaining semen trait pairs.

The genetic and phenotypic correlations among different semen traits in Landrace boars are presented in Table 7. In Landrace boars, negative genetic correlations were observed between VOL and MOT (−0.54 ± 0.24) and between VOL and CON (−0.73 ± 0.13), while a negative genetic correlation of −0.14 was found between MOT and ABN. Positive genetic correlations were observed for all remaining semen trait pairs.

## 4. Discussion

### 4.1. Heritability

Semen quality plays a crucial role in swine breeding and production [35], with poor semen quality representing a major factor leading to the culling of breeding boars [9,36]. Therefore, during boar selection and breeding, attention should be given to semen traits in addition to growth traits. The estimation of genetic parameters for these traits is important.

In this study, both single-trait and multi-trait repeatability models were used to estimate the heritability of semen traits in Large White and Landrace boars. In terms of VOL, the heritability estimates for Large White boars were 0.23 under both models, indicating a moderately heritable trait. Li et al. [22] reported that the heritability estimates for VOL in Duroc, Landrace, and Yorkshire boars ranged from 0.21 to 0.25, while Ogawa et al. [17] observed a heritability estimate of 0.29 for VOL in Duroc boars, all indicative of moderately heritable traits. In the present study, the heritability estimates for Landrace boars were 0.42 and 0.43, which are higher than those in previous reports. This discrepancy may be attributed to differences in genetic architecture between breeds, as well as differences in breeding history and selection objectives. Long-term selection practices targeting different production traits may have altered the distribution of genetic variation associated with semen volume between Large White and Landrace populations. In addition, differences in population structure and the degree of family clustering may affect the estimation of variance components, particularly in relatively small populations. The relatively limited number of Landrace boars in the present study may have reduced the information available to separate additive genetic effects from permanent environmental effects. In addition, population structure and family clustering may affect variance component estimation, especially in small populations. The limited number of Landrace boars may have increased uncertainty in the estimates. Therefore, the higher heritability estimates in Landrace pigs should be interpreted with caution.

Heritability estimates for MOT in Large White and Landrace boars ranged from 0.07 to 0.09, indicating low heritability. This is consistent with the estimate of 0.06 reported by Gruhot et al. [37] In contrast, Zhang et al. [21] reported a value of 0.29, while Li et al. [22] observed a value of 0.42 in Duroc boars, both suggestive of high heritability. For CON, the heritability estimates ranged from 0.16 to 0.22 in both breeds, representing low to moderate heritability. Wolf et al. [16] reported CON heritabilities of 0.21 and 0.18 in Large White and Landrace boars, respectively, and Wolf et al. [8] observed a range of 0.13 to 0.25 across seven different breeds, all of which are consistent with the low to moderate heritability observed in the present study. For ABN, the heritability estimates ranged from 0.07 to 0.18 in both Large White and Landrace boars, indicative of low heritability. Marques et al. [13] reported that the ABN heritability values of Landrace boars were 0.14, similar to the results of the present study, while Zhang et al. [21] found an ABN heritability of 0.29, classified as moderate and differing from the present results. These differences may arise from population structure, sample size, feeding and management practices, fixed effects, measurement protocols, and statistical models, all of which should be carefully considered in breeding programs and further validated using larger samples. In addition, because the data originated from a single breeding population and were collected through four AI stations, the estimated genetic parameters should be generalized to other commercial populations with caution.

For ABN, we found it may have moderate repeatability but low heritability. Because repeatability includes both additive genetic and permanent environmental variance. It may indicate that repeated phenotypic measurements can provide relatively stable individual information, while genetic selection based on a single record may still be inefficient for longitudinal data such as semen quality traits.

Random regression models (RRMs) can flexibly fit functions and characterize covariance structures and therefore have the potential to capture dynamic changes in genetic effects associated with continuous variables and the partitioning of variance components. Theoretically, when the model is correctly specified and sufficient data are available, RRMs may provide more accurate, unbiased, and informative estimates of heritability. However, few studies have applied RRMs to estimate genetic parameters for semen quality traits in pigs. To our knowledge, Hong et al. [38] applied RRMs to these traits in 2022 and found that higher-order polynomials were more suitable for permanent environmental effects, whereas lower-order polynomials were preferable for additive genetic effects. Similar findings were also reported by Prakash et al. [39]. In the present study, some traits in Large White pigs showed a similar pattern, in which the additive genetic effects of all four traits were best fitted with lower-order (0 or 1) polynomials, whereas their permanent environmental effects required higher-order (1–3) polynomials. The VOL, MOT, and ABN traits in Landrace pigs also followed this pattern. This may be because permanent environmental influences associated with management, health status, and environmental adaptability change nonlinearly with boar age, thereby requiring more flexible functions to adequately describe these changes. Therefore, the optimal polynomial order in RRMs may vary among traits, potentially reflecting differences in their genetic architecture and age-dependent patterns.

The results of the present study were not entirely consistent with those reported by Hong et al. [38]. Similarities lie in that both studies showed that the heritability of the four semen traits exhibited clear age-dependent patterns. In the present study, for some traits fitted with higher-order additive genetic regression, both additive genetic variance and heritability varied with age. For other traits where the order of additive genetic regression was zero, the additive genetic variance remained constant across all ages. Accordingly, the observed age-related changes in heritability were driven by alterations in permanent environmental variance and total phenotypic variance. Hong et al. [38] found the heritability of the four semen traits in Landrace pigs remained relatively stable across the entire age range (36 to 136 weeks). Such differences may be attributed to differences in genetic background, population size, and data structure, as well as differences in the age distributions of boars between the two studies. The age range in the present study covered younger boars (starting from 183 days) but did not extend to extremely old ages due to the limited amount of data at older ages. Such different age windows capture different physiological stages, directly affecting the fitting of additive genetic and permanent environmental effects in the random regression model, thereby leading to differences in the selection of optimal polynomial orders and the estimated heritability curves. Overall, the heritability of most semen traits in Large White and Landrace pigs tends to increase with age, but this does not mean that delayed selection will be more effective, as generation intervals, number of records, and genetic correlations across ages must also be considered. Moreover, the sample size in the present study may still be limited, which may have resulted in an artifact of overfitting. Future studies involving larger populations, more balanced age distributions, and a wider age coverage are needed to further validate the reliability of genetic parameter estimates.

### 4.2. Genetic Correlation

The estimated directions of genetic correlations among the four semen traits were generally consistent between the two breeds; however, some estimates had relatively large standard errors and should therefore be interpreted with caution. The analysis showed that VOL was negatively correlated with CON in both breeds, which was consistent with the results reported by Li et al. [22] in Duroc and Landrace pigs. MOT and ABN exhibited negative genetic correlations in both breeds, in agreement with the findings of Marques et al. [13] across five pig breeds. Meanwhile, MOT and CON showed positive genetic correlations in both breeds, which was also consistent with the results reported by Wolf et al. [16]. However, the magnitude of these correlations was not entirely the same. For example, VOL and MOT showed a low negative genetic correlation in Large White pigs (−0.03 ± 0.23) and a moderate negative genetic correlation in Landrace pigs (−0.54 ± 0.13). Previous studies have indicated that breed-specific differences in genetic correlations among traits are relatively common [8], which may be associated with differences in genetic backgrounds among breeds. Variations in genetic structure, allele frequencies of genes affecting multiple traits, as well as differences in pleiotropy and linkage disequilibrium patterns, may contribute to discrepancies in genetic correlation estimates among breeds [40]. A higher level of linkage disequilibrium may strengthen the genetic correlations between traits. Secondly, the estimated genetic parameters obtained may depend on population structure, breeding programs, feeding management systems, environmental conditions, semen collection and analysis processes, as well as the genetic composition of the study population. This study is based solely on small sample phenotype and pedigree information, without genomic data. This may limit the wider application of the results, and further validation is still needed in conjunction with larger populations and genomic information.

## 5. Conclusions

The results of this study indicated that the heritability of sperm motility (MOT) and abnormal sperm rate (ABN) was low, the heritability of sperm concentration (CON) ranged from low to moderate, and the heritability of semen volume (VOL) was moderate to high. The heritability of semen traits varied with boar age, and different trajectories were observed between the two breeds. Significant genetic correlations were detected Ttion with CON (−0.54 ± 0.13). In Landrace pigs, a stronger negative genetic correlation was observed between VOL and CON (−0.73 ± 0.13). These genetic parameters provide a theoretical basis for optimizing genetic evaluation and selection strategies for boar semen quality, particularly by considering age-dependent genetic variation during the breeding process.

## Figures and Tables

**Table 1:** Descriptive statistics of the semen characteristics of the two breeds.

Breed	Trait	Number of Records	Mean	Median	Max	Min	SD	CV (%)
LW	VOL (mL)	13,413	211.10	196.00	569.00	30.00	85.51	40.51
	MOT (%)	13,413	88.66	85.00	99.00	60.00	8.25	9.30
	CON (10^8^/mL)	13,413	3.43	3.28	7.80	0.10	1.15	33.51
	ABN (%)	13,413	8.02	6.00	47.10	0.00	5.70	71.04
LL	VOL (mL)	7747	221.21	205.00	574.00	32.00	85.93	38.85
	MOT (%)	7747	89.22	90.70	99.00	60.00	8.64	9.69
	CON (10^8^/mL)	7747	3.57	3.31	8.70	0.10	1.35	37.77
	ABN (%)	7747	7.19	5.80	52.40	0.00	4.91	68.26

Note: LW: Large White; LL: Landrace; VOL: semen volume; MOT: sperm motility; CON: sperm concentration; ABN: abnormal sperm rate.

**Table 2:** Heritabilities and standard errors of semen traits in Large White and Landrace boars as determined by the single-trait repeatability model.

Breed	Trait	$\sigma_{a}^{2}$ ± SE	$\sigma_{\mathrm{pe}}^{2}$ ± SE	$\sigma_{e}^{2}$ ± SE	h^2^ ± SE	r_e_ ± SE
LW	VOL (mL)	1085.41 ± 275.98	1181.51 ± 215.36	2479.85 ± 31.84	0.23 ± 0.05	0.48 ± 0.02
	MOT (%)	0.84 ± 0.32	1.80 ± 0.29	6.63 ± 0.09	0.09 ± 0.03	0.28 ± 0.02
	CON (10^8^/mL)	0.25 ± 0.06	0.20 ± 0.04	0.70 ± 0.01	0.22 ± 0.05	0.39 ± 0.02
	ABN (%)	0.07 ± 0.04	0.30 ± 0.04	0.57 ± 0.01	0.07 ± 0.04	0.39 ± 0.02
LL	VOL (mL)	2168.16 ± 651.85	640.10 ± 418.36	2350.42 ± 40.52	0.42 ± 0.10	0.54 ± 0.03
	MOT (%)	0.48 ± 0.25	0.82 ± 0.23	7.18 ± 0.12	0.06 ± 0.03	0.15 ± 0.02
	CON (10^8^/mL)	0.21 ± 0.09	0.25 ± 0.08	0.88 ± 0.02	0.16 ± 0.07	0.34 ± 0.03
	ABN (%)	0.20 ± 0.08	0.20 ± 0.06	0.75 ± 0.01	0.18 ± 0.07	0.35 ± 0.03

Note: LW: Large White; LL: Landrace; VOL: semen volume; MOT: sperm motility; CON: sperm concentration; ABN: abnormal sperm rate. $\sigma_{a}^{2}$ is the additive genetic variance, $\sigma_{\mathrm{pe}}^{2}$ is the variance of permanent environmental effects, $\sigma_{e}^{2}$ is the residual variance, h^2^ is the heritability, r_e_ is the repeatability, and SE is the standard error.

**Table 3:** Variance components and heritability values of semen traits in Large White and Landrace boars, as determined by the multi-trait repeatability model.

Breed	Trait	$\sigma_{a}^{2}$ ± SE	$\sigma_{\mathrm{pe}}^{2}$ ± SE	$\sigma_{e}^{2}$ ± SE	h^2^ ± SE	r_e_ ± SE
LW	VOL (mL)	1089.72 ± 278.66	1174.94 ± 220.26	2480.01 ± 31.85	0.23 ± 0.05	0.48 ± 0.02
	MOT (%)	0.76 ± 0.32	1.90 ± 0.30	6.63 ± 0.09	0.08 ± 0.03	0.29 ± 0.02
	CON (10^8^/mL)	0.25 ± 0.06	0.19 ± 0.04	0.70 ± 0.01	0.22 ± 0.05	0.39 ± 0.02
	ABN (%)	0.08 ± 0.04	0.28 ± 0.04	0.57 ± 0.01	0.09 ± 0.04	0.39 ± 0.02
LL	VOL (mL)	2253.16 ± 648.54	577.92 ± 407.94	2350.53 ± 40.52	0.43 ± 0.10	0.55 ± 0.03
	MOT (%)	0.54 ± 0.25	0.77 ± 0.22	7.18 ± 0.12	0.06 ± 0.03	0.15 ± 0.02
	CON (10^8^/mL)	0.27 ± 0.10	0.20 ± 0.08	0.88 ± 0.02	0.20 ± 0.07	0.35 ± 0.03
	ABN (%)	0.20 ± 0.08	0.20 ± 0.06	0.75 ± 0.01	0.17 ± 0.07	0.35 ± 0.03

Note: LW: Large White; LL: Landrace; VOL: semen volume; MOT: sperm motility; CON: sperm concentration; ABN: abnormal sperm rate. $\sigma_{a}^{2}$ is the additive genetic variance, $\sigma_{\mathrm{pe}}^{2}$ is the variance of permanent environmental effects, $\sigma_{e}^{2}$ is the residual variance, h^2^ is the heritability, r_e_ is the repeatability, and SE is the standard error.

**Table 4:** AIC and BIC values for the optimal order models of Large White and Landrace boars.

Breed	Trait	Optimal Order		AIC	BIC
		Additive Genetic Variance	Permanent Environmental Variance		
LW	VOL (mL)	1	2	118,632.19	118,707.23
	MOT (%)	0	1	40,422.70	40,460.22
	CON (10^8^/mL)	0	1	10,375.98	10,413.50
	ABN (%)	1	3	7033.95	7139.00
LL	VOL (mL)	0	1	68,056.96	68,091.74
	MOT (%)	1	2	23,733.33	23,802.88
	CON (10^8^/mL)	1	0	7826.38	7861.15
	ABN (%)	0	1	6227.93	6262.71

Note: LW: Large White; LL: Landrace; VOL: semen volume; MOT: sperm motility; CON: sperm concentration; ABN: abnormal sperm rate.

**Table 5:** Heritability ranges and standard errors of random regression models for semen traits of Large White and Landrace boars.

	LW		LL	
Trait	h^2^	SE	h^2^	SE
VOL (mL)	0.24–0.53	0.05–0.11	0.08–0.31	0.03–0.11
MOT (%)	0.05–0.08	0.02–0.04	0.02–0.15	0.03–0.07
CON (10^8^/mL)	0.13–0.23	0.03–0.05	0.26–0.53	0.06–0.08
ABN (%)	0.03–0.31	0.03–0.11	0.12–0.17	0.05–0.07

Note: LW: Large White; LL: Landrace; VOL: semen volume; MOT: sperm motility; CON: sperm concentration; ABN: abnormal sperm rate. SE: the overall standard error.

**Table 6:** Genetic and phenotypic correlations of semen quality traits in Large White boars.

Trait	VOL	MOT	CON	ABN
VOL	0.23 (0.05)	−0.01	−0.33	−0.03
MOT	−0.03 (0.23)	0.08 (0.03)	0.31	−0.15
CON	−0.54 (0.13) **	0.34 (0.19)	0.22 (0.05)	0.05
ABN	0.04 (0.25)	−0.16 (0.31)	0.29 (0.23)	0.09 (0.04)

Note: 1. VOL: semen volume; MOT: sperm motility; CON: sperm concentration; ABN: abnormal sperm rate. 2. The diagonal represents heritability. Below the diagonal represents genetic correlation coefficient, and above the diagonal represents phenotypic correlation coefficient. The standard error is indicated in parentheses. 3. ** represents a significant difference (*p* < 0.01).

**Table 7:** Genetic and phenotypic correlations of semen traits in Landrace boars.

Trait.	VOL	MOT	CON	ABN
VOL	0.43 (0.10)	−0.08	−0.41	−0.02
MOT	−0.54 (0.24) *	0.06 (0.03)	0.35	−0.11
CON	−0.73 (0.13) **	0.42 (0.25)	0.20 (0.07)	0.03
ABN	0.01 (0.25)	−0.14 (0.31)	0.10 (0.28)	0.17 (0.07)

Note: 1. VOL: semen volume; MOT: sperm motility; CON: sperm concentration; ABN: abnormal sperm rate. 2. The diagonal represents heritability. Below the diagonal represents genetic correlation coefficient, and above the diagonal represents phenotypic correlation coefficient. The standard error is indicated in parentheses. 3. * represents a significant difference (*p* < 0.05); ** *p* < 0.01.

## Data Availability

The original contributions presented in this study are included in the article. Further inquiries can be directed to the corresponding author.

## Abbreviations

The following abbreviations are used in this manuscript:

LW	Large White
LL	Landrace
VOL	semen volume
MOT	sperm motility
CON	sperm concentration
ABN	abnormal sperm rate
